# Corneal reflections and skin contrast yield better memory of human and virtual faces

**DOI:** 10.1186/s41235-022-00445-y

**Published:** 2022-10-18

**Authors:** Julija Vaitonytė, Maryam Alimardani, Max M. Louwerse

**Affiliations:** Department of Cognitive Science and Artificial Intelligence, Tilburg University, Dante Building D 134, Warandelaan 2, 5037 AB Tilburg, The Netherlands

**Keywords:** Face memory, Face perception, Virtual faces, Corneal reflections, Skin contrast

## Abstract

Virtual faces have been found to be rated less human-like and remembered worse than photographic images of humans. What it is in virtual faces that yields reduced memory has so far remained unclear. The current study investigated face memory in the context of virtual agent faces and human faces, real and manipulated, considering two factors of predicted influence, i.e., corneal reflections and skin contrast. Corneal reflections referred to the bright points in each eye that occur when the ambient light reflects from the surface of the cornea. Skin contrast referred to the degree to which skin surface is rough versus smooth. We conducted two memory experiments, one with high-quality virtual agent faces (Experiment 1) and the other with the photographs of human faces that were manipulated (Experiment 2). Experiment 1 showed better memory for virtual faces with increased corneal reflections and skin contrast (rougher rather than smoother skin). Experiment 2 replicated these findings, showing that removing the corneal reflections and smoothening the skin reduced memory recognition of manipulated faces, with a stronger effect exerted by the eyes than the skin. This study highlights specific features of the eyes and skin that can help explain memory discrepancies between real and virtual faces and in turn elucidates the factors that play a role in the cognitive processing of faces.

## Introduction

Humans are highly visually oriented species (Van Essen, 2004), with one type of visual stimulus especially capturing our attention—the face (Hershler & Hochstein, 2005). This is not surprising as face processing plays an important role in human communicative interactions (Hernández-Gutiérrez et al., 2021). Superior face processing skills in humans can be linked to highly specialized neural circuitry (Duchaine & Yovel, 2015), as well as the high variability in facial morphology, particularly in comparison to other species (Sheehan & Nachman, 2014).

Given the specialized neuro-cognitive mechanisms for human face processing, it becomes increasingly relevant to understand the extent to which human face processing extends to the faces of entities emulating the appearance of humans, for instance, Intelligent Virtual Agents (IVAs). IVAs are embodied virtual characters that can interact with humans using verbal, para-verbal, and nonverbal behaviors (Lugrin, 2021). With advances in computer graphics (Alexander et al., 2009), the faces of IVAs have become photorealistic (Seymour et al., 2017). The growing prevalence of using IVAs as stimuli in research (Kätsyri et al., 2020) and the interest to use them in different use cases, such as e-commerce (Etemad-Sajadi, 2016), healthcare (Robinson et al., 2014), and education (Belpaeme et al., 2018) highlight the importance of investigating the similarities between processing IVA faces, henceforth, virtual faces and natural human faces.

Despite the progress in computer-generated imagery, the processing of virtual faces is different than the processing of natural human faces. Previous perception research revealed that computer-generated faces are generally distinguishable from natural faces (Farid & Bravo, 2012; Vaitonytė et al., 2021), and that information across the whole face, the eyes, and the skin, is employed to make this distinction (Balas & Tonsager, 2014). Relatedly, the fidelity of specific features in the eyes and the skin (as discussed in detail below) has been recently found to be responsible for identifying virtual agent faces as virtual rather than human-like (Vaitonytė et al., 2021).

Memory studies, too, point to a processing discrepancy between computer-generated and human faces (Balas & Pacella, 2015; Crookes et al., 2015; Kätsyri, 2018). Balas and Pacella (2015) used real photographs and identity-matched computer-generated counterparts, created using FaceGen software, which allows importing a frontal and two lateral photographs of the face to generate an individualized avatar. Balas and Pacella (2015) found that real faces were significantly better remembered than computer-generated faces. Crookes et al. (2015) who used real photographs of Caucasian and Asian faces and computer-generated counterparts, as well as computer-generated Caucasian and Asian faces generated at random using FaceGen, reached the same conclusion, i.e., higher memory recognition accuracy was found for natural than computer-generated faces. Crookes et al. (2015) also showed that the Other Race effect (ORE; better face memory for one’s own race than other races) was reduced for computer-generated faces compared to real facial photographs.

Similarly, Kätsyri (2018) employed real photographs and virtual faces that were generated in FaceGen and that were also matched on low-level features (global luminosity and spatial frequency contents). Virtual faces matched on low-level visual characteristics were still recognizable as virtual. Regarding participant memory, while Kätsyri (2018) found that the sensitivity index *d'* was not higher for real faces than virtual faces, the response bias index *c* was higher for virtual faces, indicating participants found that virtual faces were more similar to one another than real human faces were. It was previously suggested that computer-generated faces lack discriminating information in the form of fine-grained surface texture (Crookes et al., 2015). This prediction is compatible with face recognition literature, showing that when spatial frequency information is reduced (where spatial frequencies refer to luminance variations, with high spatial frequencies encoding fast luminance variations and hence more detail), face recognition accuracy also drops (Sandford et al., 2018). However, while the heterogeneity of facial details may be important for remembering virtual faces as it is with natural faces, this prediction has not been directly tested.

Vaitonytė et al. (2021) showed both experimentally and computationally that the intricacy of facial details is important for the perceived human-likeness, allowing perceivers to distinguish between natural human faces and virtual faces. The specific features that were indicative of the face being virtual were *skin contrast*, i.e., the degree to which skin texture is rough versus smooth, and *corneal reflections*, i.e., the white foci in each eye that occur when the ambient light reflects from the surface of the cornea. Reductions in skin contrast and corneal reflections caused one to perceive the face low in human-likeness.

The reasons behind the predicted influence of the skin contrast and corneal reflections are based on the previous literature that reported the features affecting face recognizability, i.e., spatial frequency information (Sandford et al., 2018) and contrast polarity (Gilad et al., 2009). Reducing spatial frequency information negatively affects face recognition accuracy because the face loses detail (Sandford et al., 2018), whereas the potential role of corneal reflections might be associated with the broader role that contrast polarity relationships play in face recognition (Gilad et al., 2009). Contrast polarity relationships refer to the eyes being darker than the forehead and the cheeks, known to be a remarkably stable feature; if these relationships are reversed, for instance, by applying contrast negation, the face recognition becomes impaired. According to Gilad et al. (2009), contrast polarity relationships may be important for typical face processing, in that they represent regularities in the data that get incorporated by the visual system when it learns about visual objects. It is unclear how regular the presence of corneal reflections is. Corneal reflections might be less regular than contrast polarity relations across the face because depending on the lighting conditions, corneal reflections may be pronounced or reduced. However, one may argue that corneal reflections are a feature that gets incorporated into face representations by the visual system.

In the current study, we examined the extent to which the previously identified features of skin contrast and corneal reflections impacted the memory of different faces. We tapped into more general cognitive processes by asking participants to remember stimuli. In day-to-day life, people commonly encounter situations that require remembering different faces while assessing human-likeness is arguably infrequent. We examined the role of skin contrast and corneal reflections in memory by conducting two experiments. Experiment 1 had high-quality virtual faces collected “in the wild,” while Experiment 2 used human faces obtained from a picture database that were further manipulated to reduce skin contrast and corneal reflections. Although both experiments were conducted with the same group of participants, they can be best understood as independent experiments due to the different nature of stimuli. Dawel et al. (2021) argued that an approach that seeks convergent evidence using a set of controlled images and a set of images collected “in the wild” is valuable when studying face processing. Our approach is, however, not amenable to directly comparing the memory recognition performance in virtual faces versus human faces, rather we sought to more generally understand whether the predicted features were of influence on memory.

## Experiment 1

Experiment 1 used high-quality virtual faces available from different companies that employ cutting-edge techniques (i.e., 3D scanning) to test how facial details present in virtual faces were associated with participant face memory performance. Following Vaitonytė et al. (2021), we predicted that the virtual faces with higher skin contrast and a higher number of corneal reflections would be remembered better compared to the virtual faces with lower skin contrast (i.e., smoother skin) and fewer corneal reflections.

### Method

#### Participants

Sixty-three students at Tilburg University (34 females, 28 males, and one person who preferred not to indicate gender, Age: *Mean *= 22.65, *SD* = 4.19) took part in the experiment in exchange for either partial course credits or a candy bar. The majority of participants identified with Caucasian ethnicity (*n* = 42), followed by Asian (*n* = 9), Black (*n* = 2), Hispanic (*n* = 2), and Middle Eastern (*n* = 2) ethnicities, while 6 participants indicated “Other.” Participants were recruited via the university participant pool and advertisements put on campus. The experiment was approved by the Research Ethics and Data Management Committee of the Tilburg School of Humanities and Digital Sciences (identification code: REC#2019/03). All participants provided consent prior to their participation in the experiment.

#### Stimuli

Experimental stimuli consisted of photorealistic virtual agent faces (*n* = 24, 12 female), collected from the Internet using the following criteria: (1) the photographs of the virtual face had to be of high quality, (2) the face had to be presented in frontal view, and (3) the face was not covered with hair that obscured facial features. No changes were made to the facial images of virtual faces. We used searches “digital human” and “digital humans” with the aim to collect virtual faces created by companies that work in the realm of “digital human” technology. These companies use techniques such as 3D scanning and/or deep neural networks, which permit creating photorealistic faces. To prepare stimuli, virtual faces were cropped to an oval removing all non-facial information (e.g., hair), with a slightly varying width due to inherent variation in the facial width (from 550 to 650 pixels) and a constant height (800 pixels). By collecting virtual agent faces, we aimed to obtain a sample of photorealistic virtual faces, and also have a more spontaneous set of faces. Such stimulus set can be considered similar to the face photographs collected “in the wild” that are sometimes used in studies on face recognition, whereby e.g., lighting conditions or face age may differ among images.

#### Procedure

Participants received instructions both in writing and verbally. First, participants were presented with written instructions, after which they signed an informed consent digitally and filled in a demographic questionnaire. The written instructions, the informed consent, and the questionnaire were presented in Qualtrics (Qualtrics, 2021). Following this, the experimenter explained the task in Experiment 1 and Experiment 2 verbally. We combined the presentation of instructions for Experiment 1 with those for Experiment 2 since one experiment followed another, with a short break in-between. The decision against combining the virtual faces and the human faces from Experiment 2 into a single experiment was based on the two classes of images forming clearly distinct groups.

Participants were told that they would see a series of virtual agent faces that needed to be memorized (henceforth “study” phase). Next, they would get statements whose veracity needed to be judged (distractor task), and they would then again see a set of virtual agent faces and would decide whether they had previously seen each face or not (henceforth “test” phase). There was no time limit in “test” phase, but participants were asked to use their immediate judgment to make decisions. Participants were asked to use their index fingers to press the *M* key if the face was “old” (presented previously) or a *Z* key if the face was “new” (presented for the first time). The distractor task between “study” and “test” included general knowledge statements (e.g., “Monaco is the smallest country in the world”), for which participants selected whether they thought the statement was true or false. The duration of completing the distractor task slightly varied across participants, because it depended on each participant’s speed, but on average it lasted approximately 2–3 min. Neither answers nor response times in the distractor task were relevant or used for the analysis.

**Fig. 1:**
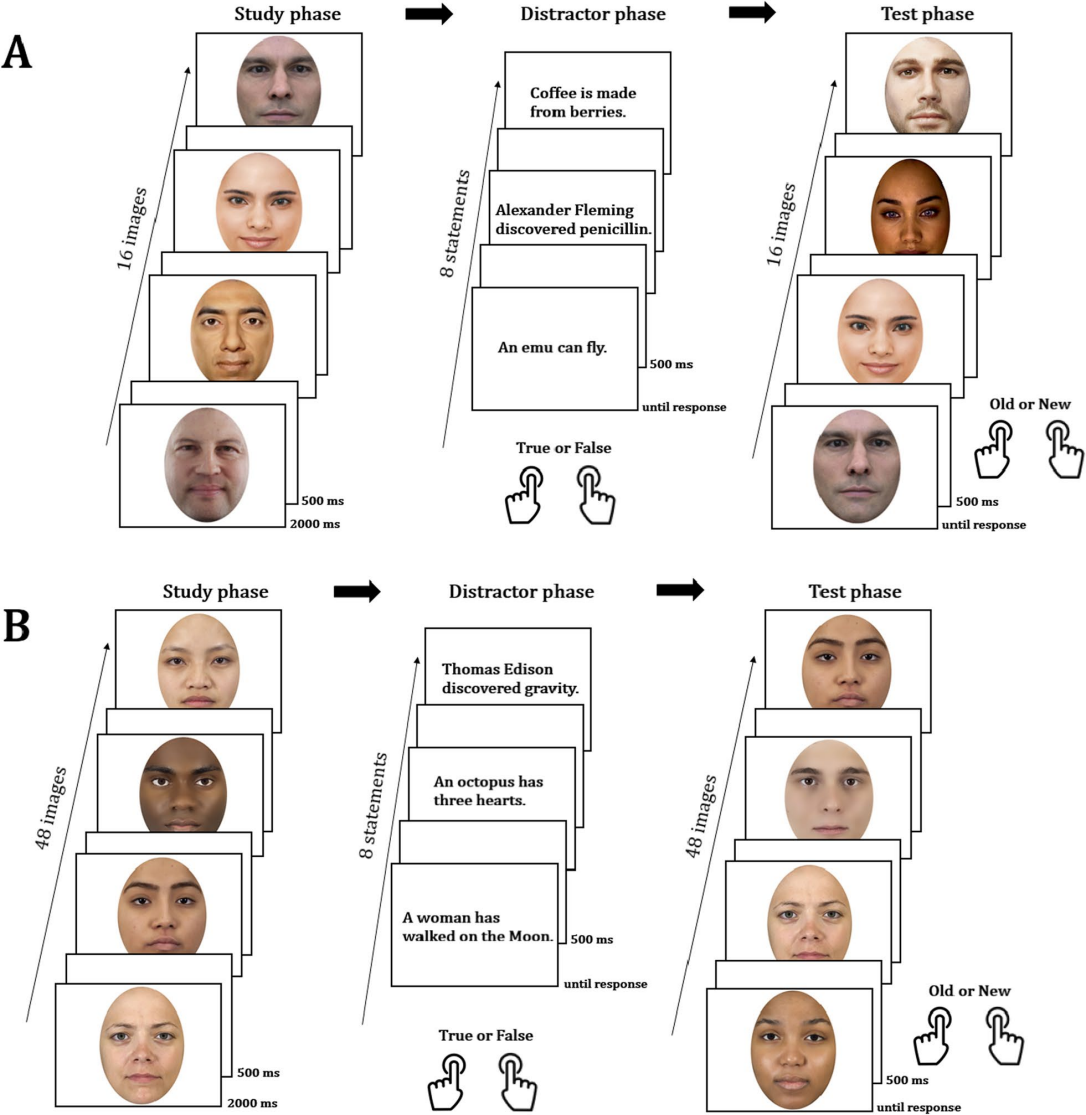
Schematic overview of the experimental procedure in **A** Experiment 1 and **B** Experiment 2. In the “study” phase, participants saw each face presented on the screen for 2000 ms followed by a blank screen that was presented for 500 ms. In the subsequent distractor phase, participants read statements and indicated their veracity. Finally, memory was tested in the “test” phase by presenting half of the earlier presented images in addition to new facial images

Stimuli were presented using the PsychoPy software (Peirce, 2007) on a laptop (Dell E5480) with a screen resolution of 1920 × 1080. Participants sat approximately 40 cm from the screen. Four face stimuli, one virtual face, and three human faces, none of which were part of the experimental stimuli, were given to participants as practice. Having finished the practice trials, participants started Experiment 1, in which they saw 16 virtual faces (half female) (Fig. 1A) presented for two seconds following Schyns et al. (2002). Following the “study” phase, participants read eight statements and indicated their veracity. In the “test” phase, participants saw 16 virtual faces of which 8 images were previously not shown. Virtual faces to be shown to participants as “old” or “new” were selected randomly. However, due to the limited sample size of the available virtual agent faces, they were not counterbalanced. Therefore, all participants saw the same set of images as “new” and the same set of images as “old.” Participants’ accuracy and response times (RTs) were collected as dependent variables.

#### Computational measures

While we did not manipulate virtual faces, we used two computational measures, as previously described in Vaitonytė et al. (2021), to assess skin smoothness versus roughness and the presence of the corneal reflections. For the assessment of skin smoothness, we measured contrast variations in facial images, which we converted to grayscale before carrying out the calculations. The developed measure for skin, termed “skin contrast,” identified for each pixel in a facial image the biggest difference with its adjacent pixels (each pixel had 8 neighbors). A matrix of those differences in contrast quantifications had been used to derive the median for each facial image. Therefore, the output of the algorithm was the median value of skin contrast per image. In this computation, we used the whole face as input because: (1) taking the whole face versus isolated parts did not yield differing results, and (2) taking whole faces confers better generalizability. For the computational assessment of corneal reflections, we counted the white foci in each eye, selecting the iris and pupil. Each image was first converted to grayscale and then bright and dark pixels were identified. Finally, all bright pixels that were connected to each other were identified as corneal reflections, yielding the number of corneal reflections as measure.

#### Analysis

Data were preprocessed and analyzed in R (version 4.0.3; R Core Team, 2021). We transformed participant responses into sensitivity index *d*′ and the response bias index *c* in the framework of the Signal Detection Theory (SDT, Stanislaw & Todorov, 1999). In addition, we fitted Generalized Linear Mixed Models (GLMM) to raw accuracy scores and response times via the *lme4* package (Bates et al., 2015). Response times longer than three standard deviations from the mean were removed, affecting 2.15% of the data from Experiment 1. We included the SDT measures in addition to raw accuracy scores to account for the bias introduced by participants, in that in SDT, sensitivity to the task (*d*′) is measured independently of response bias (*c*).

Responses could be assigned to one of the four categories: (1) *Hits* when the face was correctly identified as old, (2) *Misses* when participants failed to categorize old faces as such, (3) *False alarms* when unseen faces were mistakenly identified as old, and (4) *Correct rejections* when unseen faces were identified as new. When calculating *d*′, transformations were applied to values of hits and false alarms of 0 using the log-linear rule (Hautus, 1995) to avoid obtaining values of positive and negative infinity. Higher *d*′ values suggested higher sensitivity, indicative of having many hits and few false alarms. The response bias index *c* determined the preferred response favored by participants, i.e., conservative or liberal. Conservative responding is represented by positive values of *c* and can be understood as the preference to respond “old,” while negative values of *c* are indicative of liberal responding, i.e., “new.” In Experiment 1, we were interested in the values of *d*′ across the virtual faces. The sensitivity index *d*′ was calculated for each virtual face (*n* = 16), i.e., averaged across participants, and for each participant (*n* = 63), i.e., averaged across virtual face images.

In the mixed-effects model analyses, we looked at whether the computational measures, i.e., the number of corneal reflections and the skin contrast score, obtained for each virtual face as described above (both treated as continuous predictors in the models), were predictive of the accuracy and the RT responses. Mixed-effects logistic regression analysis was conducted on participants’ original responses: 1 = correct and 0 = incorrect, whereby correct meant that the participant correctly remembered whether or not a face was presented earlier. Linear mixed-effects regression was conducted on RTs (measured in milliseconds). Across all analyses, participants were a random factor while we did not include items as a random factor to avoid eliminating item variance that needed to be attributed to the predictors of interest (i.e., the number of corneal reflections and the skin contrast score). Model comparisons were conducted via the likelihood ratio (LR) tests to determine the significance of predictors of interest.

### Results

#### Sensitivity index *d*′ and response bias index c

**Fig. 2:**
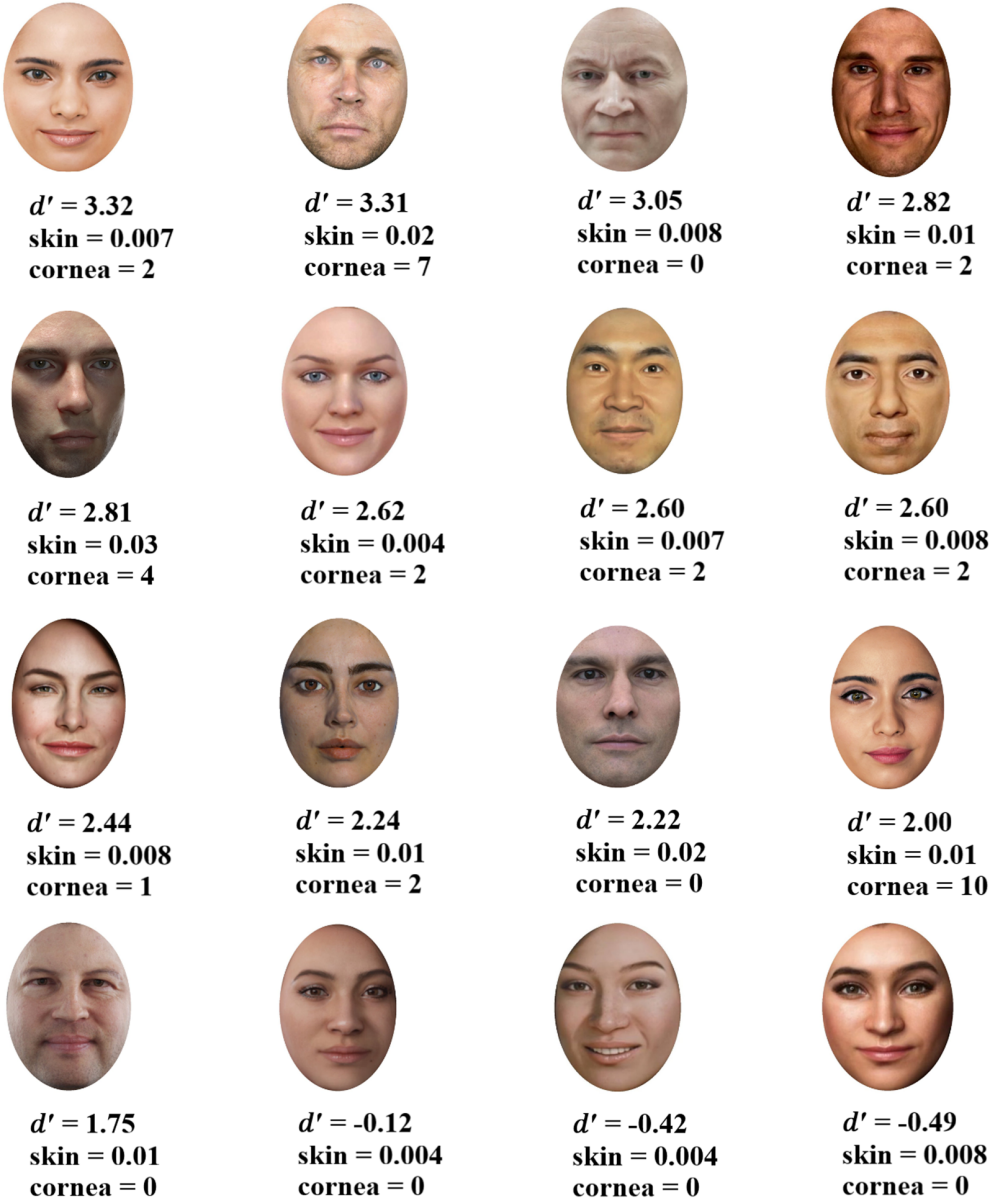
*d*′ scores averaged across participants (top row), the skin contrast value denoted as “skin” (middle row), and the number of corneal reflections denoted as “cornea” (bottom row) for each virtual face. The larger values of *d*′ show good memory, whereas the negative values indicate that the face was poorly remembered

The values of the sensitivity index *d*′ were generally high across virtual face images, with a mean of 2.05 (no sensitivity being 0). This suggests that the virtual faces were overall remembered well, with the exception of three virtual faces (bottom row in Fig. 2) yielding negative scores. Figure 2 presents the virtual faces that were employed to test memory in Experiment 1 together with the sensitivity index *d*′ and the computational measures associated with each face. A slight bias was found among participants toward responding having seen a face before (average of *c* scores = 0.63). Overall, the results of Experiment 1 suggested that participants were able to recall virtual faces well.

#### Accuracy

We first ascertained which of the models were significant using LR tests. The number of corneal reflections in the eyes was a significant predictor when compared to the null model, *χ*^2^(1) = 38.18, *p* < 0.001. When skin contrast as the predictor was added to the model, the model improved, *χ*^2^(1) = 13.94, *p* < 0.001 compared to the model that only contained the number of corneal reflections.

Overall, we found that participants were more likely to have higher memory recognition accuracy when virtual faces had a higher number of corneal reflections (*β* = 0.19, SE = 0.05, *z* = 3.64, 95% CI [0.09, 0.30], *p* < 0.001), and when the faces had increased skin contrast (*β* = 63.83, SE = 18.01, *z* = 3.54, 95% CI [29.00, 102.08], *p* < 0.001).

#### Response times

Contrary to accuracy scores, the null model for the response times compared with the model containing the number of corneal reflections as a predictor yielded no improvement in the model, *χ*^2^(1) = 2.74, *p* = 0.10. In line with the accuracy scores, adding skin contrast as a predictor did improve the model, *χ*^2^(1) = 85.25, *p* < 0.001. The model with the number of corneal reflections and skin contrast as predictors was significantly better than the model containing only the number of corneal reflections.

We found that virtual faces with increased corneal reflections yielded faster response times (*β* =  − 43.15, SE = 7.88, *t*(914.85) =  − 5.48, 95% CI [− 58.60, − 27.69], *p* < 0.001). However, higher skin contrast yielded significantly slower (not faster) response times (*β* = 31,062.75, SE = 3281.45, *t*(917.23) = 9.47, 95% CI [24621.28, 37,500.92], *p* < 0.001).

### Discussion

The results in Experiment 1 showed that across the virtual face images, the values of sensitivity index *d*′ were high, with the exception of three faces. High memory recognition accuracy in general might, however, have resulted from a relatively small number of faces to memorize. In terms of raw accuracy scores, skin contrast and corneal reflections were predictive of memory performance. The virtual faces with a higher skin contrast and a higher number of corneal reflections were more likely to be remembered better than the faces with reductions in these features. The two features thus covaried with accuracy in the hypothesized direction, i.e., facilitating memory performance. Participants responded significantly faster on the faces in which the eyes had a higher number of corneal reflections, whereas increased skin contrast yielded significantly longer response times. This difference in the direction of the observed effects on response times was not predicted. It might be that more detailed skin appearance as indicated by higher values of skin contrast might have required more processing time because skin is a more global feature (compared to corneal reflections that are more localized).

Based on these results with virtual faces, for better memory of the face, the skin texture may be suggested to be rougher (rather than smoother) and the eyes with corneal reflections included (rather than reduced). The downside of Experiment 1, however, is that we did not control for any extraneous factors (e.g., face distinctiveness). It is possible that distinctiveness, known to affect the ability to remember faces (Valentine, 1991), has covaried with the features of interest, and thus explains the results as much as those features do. A controlled set of faces is therefore needed to seek convergent evidence regarding the extent to which altering skin contrast and the presence of corneal reflections affect face memory. We address this caveat in Experiment 2.

## Experiment 2

Experiment 2 used human faces and examined how face memory was affected by the extent of manipulation made to different faces by smoothening the skin and removing/preserving the corneal reflections. We predicted that violations in the two features would negatively impact memory performance. Based on the prior literature (Vaitonytė et al., 2021), we expected that the absence of corneal reflections would exert a stronger influence, in that the worst memory would be observed with respect to the faces lacking corneal reflections, with and without changes in skin.

### Method

#### Participants

Sixty-three participants took part in this experiment, the same sample as those participants in Experiment 1.

#### Stimuli

The stimulus set consisted of the photographs of human faces that were taken from the Chicago Face Database (CFD; Ma et al., 2015), randomly selecting 12 female and 12 male individuals of four races that are present in CFD: Asian, Black, Caucasian, and Hispanic. We aimed to include the faces of different ethnicities and both sexes in the stimulus set to have a representative sample of the database. All images of the 96 different identities (i.e., (4 races × 12 female pictures) + (4 races × 12 male pictures) had a neutral facial expression, were free from makeup, and depicted the face in frontal view. The CFD images allowed us to better control for extraneous factors, i.e., distinctiveness and attractiveness. We ascertained that the selected images were comparable in perceived distinctiveness and attractiveness relying on the extensive norming data that accompanies the images present in CFD. Each face in CFD was evaluated by a large group of raters on a number of dimensions. The ratings in CFD include, for instance, trustworthiness, dominance, and competence in addition to distinctiveness and attractiveness. Moreover, various characteristics were measured from images related to face morphology, including nose shape, face roundness, width-to-height ratio, as well as low-level features (i.e., luminance). Based on the luminance values that CFD lists, we ascertained that the selected images had comparable median luminance values as measured globally from the face (*M* = 141.22, *SD* = 30.42), which is not surprising because the images in CFD were acquired under standardized conditions (i.e., the backdrop, lighting conditions, and camera settings were kept constant). There was only some variation in luminance due to ethnicity of the face, such that Black faces (*M* = 93.75, *SD* = 16.22) had slightly lower luminance values than other ethnicity faces: Asian (*M* = 160.46, *SD* = 13.85), Caucasian (*M* = 153.50, *SD* = 10.10), and Hispanic (*M* = 157.17, *SD* = 10.19). The selected faces were found to be moderately attractive (*M* = 3.34, *SD* = 0.61), and there was not much variation across different ethnicities regarding perceived attractiveness: Asian (*M* = 3.21, *SD* = 0.56), Black (*M* = 3.33, *SD* = 0.43), Caucasian (*M* = 3.39, SD = 0.80), and Hispanic (*M* = 3.44, *SD *= 0.59). Similarly, the raters assessed each face on how unusual it was, i.e., whether it would stand out in the crowd which is comparable to distinctiveness. The selected stimuli were low in distinctiveness (*M* = 2.24, *SD* = 0.40). There was not much variation across different ethnicities regarding perceived distinctiveness: Asian (*M* = 2.17, *SD* = 0.39), Black (*M* = 2.39, *SD* = 0.30), Caucasian (*M* = 2.24, *SD* = 0.36), and Hispanic (*M* = 2.14, *SD* = 0.51).

**Fig. 3:**
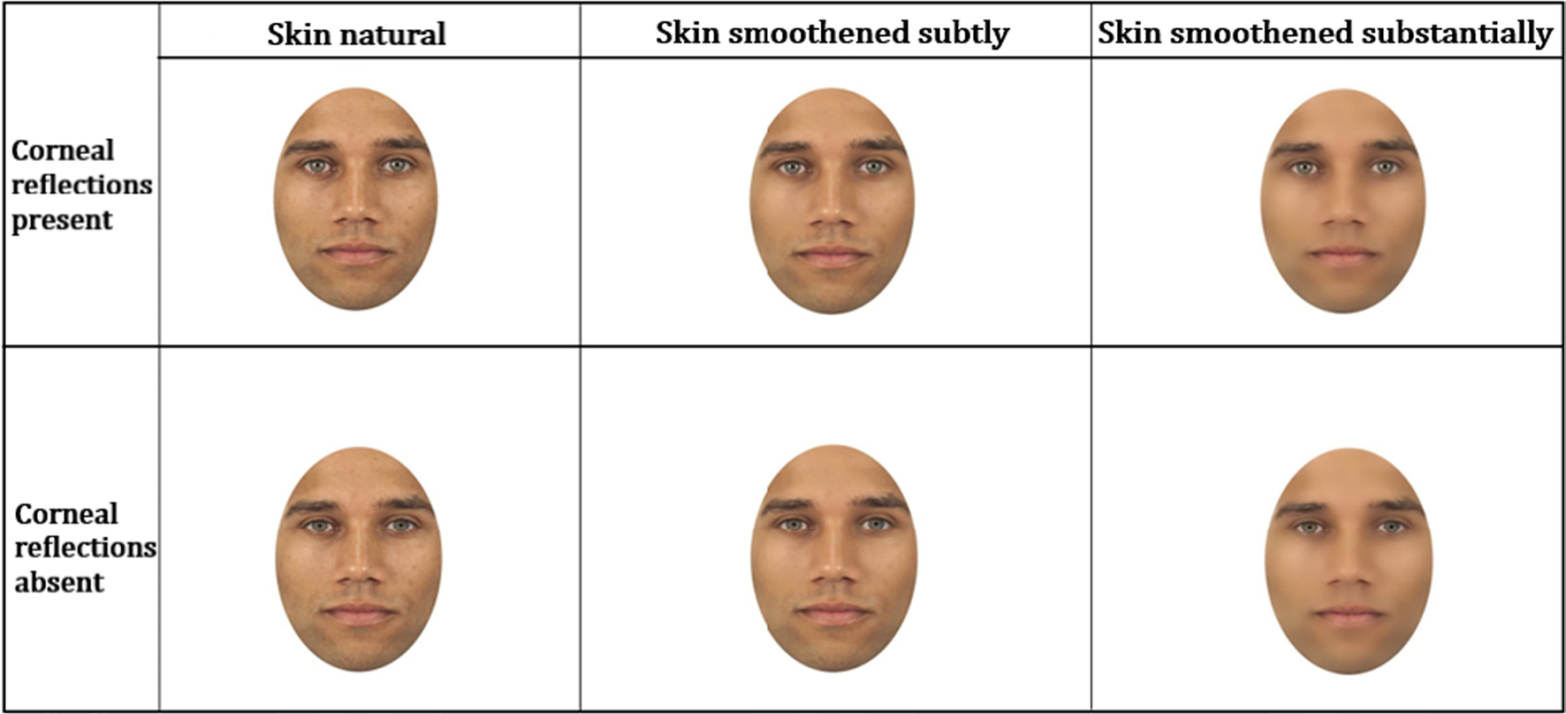
Example of a face stimulus in all conditions in Experiment 2. The top row shows different levels of skin contrast: the face with the smoothest skin (right), the face with skin that is the least smooth (left), and the face whose skin is in-between regarding smoothness (middle) when corneal reflections are present. The bottom row shows each face with differing skin appearance when corneal reflections are removed

To alter the original CFD faces, we applied manipulations to the eyes and the skin of the selected faces, whereby a 2 (corneal reflections present vs. corneal reflections absent) × 3 (skin natural vs. skin smoothened subtly vs. skin smoothened substantially) design was used, yielding six different sets of stimuli. In sum, the experimental stimuli comprised the faces that represented 2 sexes (female, male) × 4 races (Asian, Black, Caucasian, Hispanic) × 6 conditions (the corneal reflection and the skin manipulations). We used a within-subjects design since each participant was exposed to all manipulations in the eyes and the skin as shown in Fig. 3, but the same participant was never exposed to a specific face more than once. This means that we created six different lists with different randomized order of items, e.g., while one participant was presented with a Caucasian male face with the corneal reflections removed, the other participant saw the same Caucasian male face with the corneal reflections removed and the skin smoothened subtly. This way the same identity of the face was never presented more than once to the same participant.

To prepare the images, we cropped them to an oval to remove all non-facial information (e.g., hair). While the width slightly varied due to inherent variation in the facial width (from 550 to 650 pixels), the height was kept constant (800 pixels). Manipulations in the eyes and skin of humans were made using the Adobe software (Lightroom, version 1.10.0.4 and Photoshop, version 13.0.1 × 32). To smoothen the skin for subtle skin smoothness, a filter was applied across the entire face while the facial areas that contained eyebrows, eyes, and lips were preserved. The filter was created based on a preset of fixed parameters (luminance = 50 and noise = 100), which were set through experimentation in order to create the skin that was smooth, but not too silky. To create faces with substantial skin smoothness, the same filter was applied twice. The removal of the corneal reflections consisted of selecting the white foci in both the right and left eyes and replacing them with the areas next to them (i.e., the color of the iris). The rationale to smoothen the skin so as to create two different degrees of smoothness was such that we aimed to have a fine-grained manipulation. Had we only manipulated skin in a subtle manner, such change might have been too subtle. Similarly, manipulating skin in a substantial manner only might have created a sharp distinction between natural faces and manipulated faces.

#### Procedure

Participants commenced Experiment 2 after a short break following the completion of Experiment 1. The general procedure of Experiment 2 was the same as in Experiment 1 and is shown in Fig. 1B. Participants first memorized the faces in the “study” phase, then they received a set of statements whose veracity they needed to indicate, after which there followed the “test” phase where participants decided whether or not each presented face had been shown before. In the “study” phase of Experiment 2, participants were presented with a total of 48 faces, each presented for 2 s, half of which were presented in the “test” phase. The other 24 faces comprised a set of new (previously not shown) faces, including the natural faces and the items in which the eyes and the skin were manipulated as described above. The distractor task was similar to Experiment 1. The order of items was pseudo-randomized. The duration of completing the distractor task slightly varied across participants, depending on each participant’s pace, but on average lasted about 2–3 min.

The decision to present 48 faces in the “study” and “test” phases was based on a pilot test which showed that presenting 48 faces across the 6 conditions (8 images per condition) provided a suitable balance between having a sufficiently high number of items per condition and preventing subject fatigue. As in Experiment 1, participants’ accuracy and RTs were collected as dependent variables.

#### Analysis

Data were preprocessed and analyzed in R (version 4.0.3; R Core Team, 2021). Participant responses were transformed into sensitivity index *d*′ and the response bias index *c* in the SDT framework (Stanislaw & Todorov, 1999), while raw accuracy and RTs were fitted with GLMM. We compared the values of *d*′ between different conditions using the Mann–Whitney *U* tests (as *d*′ values were not normally distributed). The significance threshold was adjusted using the Bonferroni correction method (*p* < 0.05/15 = 0.003) based on the fifteen comparisons between 2 × 3 conditions (the corneal reflection and the skin manipulations).

In the Mixed-Effects Model analyses, we looked separately at the effect of the skin conditions (skin natural vs. skin smoothened subtly vs. skin smoothened substantially) and the eye conditions (corneal reflections present vs. corneal reflections absent) on accuracy and RTs. The reference categories were “skin natural” and “corneal reflections present” regarding the skin and the eyes, respectively. Both participants and items were included as random factors in all models. We thus looked at the effect of each predictor on its own so as to be able to understand how the absence of corneal reflections versus the smoothening of the skin impacted memory performance. We preprocessed the response time data by removing RTs longer than three standard deviations from the mean both by participant and by condition, affecting 2.68% of the data.

### Results

#### Sensitivity index *d*′ and response bias index *c*

We compared 2 × 3 conditions with one another using the Mann–Whitney *U* tests, which resulted in fifteen comparisons and an alpha level of 0.003. We found that only in a few cases the results survived the *p*-value correction. Tables 1 and 2 present an overview of the results. As can be seen in Table 2, compared to original faces (corneal reflections present and skin natural), memory was significantly worse for faces with corneal reflections absent and skin natural (*p* < 0.001). Similarly, when compared to the original faces, memory was worse for the faces with corneal reflections absent and skin smoothened subtly (*p* < 0.001), as well as the faces with corneal reflections absent and skin smoothened substantially (*p* < 0.001). There was a tendency for the faces with corneal reflections present and skin smoothened substantially to be remembered worse than the original faces (*p* = 0.004). Similarly, there was a tendency for the faces with corneal reflections present and skin smoothened subtly to be remembered worse than the original faces (*p* = 0.009). For all other comparisons, having applied the Bonferroni correction, the results of memory differences turned out to be not statistically significant. Overall, this means that the removal of corneal reflections significantly reduced the memory recognition accuracy while the smoothening of skin did not have such an effect, albeit a tendency.

**Table 1:** Response times, accuracy, sensitivity index *d*′ and response bias *c* across different conditions

Condition	Mean RT (SD) in ms	Mean accuracy (SD) in %	Mean value of *d*′	Mean value of *c*
Corneal reflections present and skin natural	1360 (691)	70.49 (45.65)	0.68	0.78
Corneal reflections present and skin smoothened subtly	1318 (688)	64.10 (48.02)	0.45	0.64
Corneal reflections present and skin smoothened substantially	1286 (661)	63.78 (48.11)	0.45	0.67
Corneal reflections absent and skin natural	1393 (707)	55.13 (49.79)	0.19	0.67
Corneal reflections absent and skin smoothened subtly	1350 (674)	57.35 (49.51)	0.24	0.62
Corneal reflections absent and skin smoothened substantially	1379 (705)	54.45 (49.85)	0.15	0.75

**Table 2:** All binary comparisons of *d*′ values between different conditions with the associated effect size

		Corneal reflections present and skin natural	Corneal reflections absent and skin natural	Corneal reflections present and skin smoothened subtly	Corneal reflections absent and skin smoothened subtly	Corneal reflections absent and skin smoothened substantially	Corneal reflections present and skin smoothened substantially
Corneal reflections present and skin natural	*r, p*-value		0.43, < 0.001*	0.23, 0.009	0.38, < 0.001*	0.44, < 0.001*	0.25, 0.004
Corneal reflections absent and skin natural	*r, p*-value			0.24, 0.008	0.05, 0.59	0.02, 0.85	0.23, 0.01
Corneal reflections present and skin smoothened subtly	*r, p*-value				0.18, 0.04	0.24, 0.008	0.02, 0.85
Corneal reflections absent and skin smoothened subtly	*r, p*-value					0.06, 0.48	0.18, < 0.05
Corneal reflections absent and skin smoothened substantially	*r, p*-value						0.22, 0.01
Corneal reflections present and skin smoothened substantially							

*r* denotes the effect size, *p < 0.003 is significant after applying the Bonferroni correction

#### Accuracy

As shown in Table 1, participants were most accurate at remembering original faces (70.49%), while they were least accurate on the faces with corneal reflections absent and skin smoothened substantially (54.45%), the faces with corneal reflections absent and skin natural (55.13%), and the faces with corneal reflections absent and skin smoothened subtly (57.35%). The two conditions in which manipulations to the face were made only regarding the skin, i.e., corneal reflections present and skin smoothened subtly (64.10%), and corneal reflections present and skin smoothened substantially (63.78%), were only slightly worse remembered compared to original faces with corneal reflections present and the skin that was natural.

We investigated the observed tendencies in participant ability to recall different faces by fitting the accuracy data to Mixed-Effects Logistic Regression analysis and using the LR tests to assess the model fit. We first added eyes to the model as the predictor, which produced a better fit to the data compared to the null model, *χ*^2^(1) = 11.49, *p* < 0.001. When skin as the predictor was added to the model, it did not improve it, *χ*^2^(2) = 1.63, *p* = 0.44. The eyes-by-skin interaction also did not produce a better fit to the data when compared to the model containing only eyes as the predictor, *χ*^2^(4) = 2.48, *p* = 0.65.

The effect of eyes suggested that the faces that had no corneal reflections (*β* =  − 0.47, SE = 0.14, *z* =  − 3.45, 95% CI [− 0.75, − 0.20], *p* < 0.001) were recalled significantly worse compared to the faces that had corneal reflections present. On the other hand, participants’ memory of the faces that had skin smoothened subtly was not significantly different from the faces that had natural skin (*β* = 0.06, SE = 0.18, *z* = 0.33, *p* = 0.74), nor was their memory worse with the faces that had skin smoothened substantially (*β* =  − 0.15, SE = 0.17, *z* =  − 0.89,  *p* = 0.37).

These results indicated that removing corneal reflections from the eyes was detrimental to memory performance, while if only the skin was smoothened, subtly and substantially, this did not affect memory performance. However, as can be seen from the coefficient values, substantially smoothening the skin affected memory performance in the expected direction; such faces tended to be remembered worse, but not significantly so.

#### Response times

As shown in Table 1, participants tended to respond the slowest on items that had changes made to the eyes only, i.e., the faces with corneal reflections absent and skin natural (1393 ms) whereas they were the fastest with faces that had corneal reflections present and skin smoothened substantially (1286 ms). Although these tendencies were not statistically significant, we reported the results for completeness.

When we compared the null model with the model containing eyes as the predictor, it did not produce a better fit to the data, *χ*^2^(1) = 3.50, *p* = 0.06. Adding skin as predictor to the model did not improve it, *χ*^2^(2) = 1.55, *p* = 0.46, nor did the interaction term, *χ*^2^(5) = 7.80, *p* = 0.17. Participants responded equally fast to the faces that had the eyes with corneal reflections present and the faces that had the eyes with corneal reflections absent (*β* = 51.92, SE = 27.53, *t*(72.87) = 1.87, *p* = 0.06). Across the skin conditions, participants’ response time to faces with skin smoothened subtly (*β* =  − 36.04, SE = 33.71, *t*(67.51) =  − 1.07, *p* = 0.29) and faces with skin smoothened substantially (*β* =  − 35.80, SE = 33.65, *t*(67.32) =  − 1.06, *p* = 0.29) was shorter but not significantly different from response times on the faces with natural skin. As indicated by the negative coefficient values, participants tended to speed up for faces whose skin was smoothened.

### Discussion

The results in Experiment 2 showed decreased memory recognition performance for faces that lacked corneal reflections; however, the smoothening of the skin did not affect memory performance. Participants’ response time was not affected by the manipulations made to the skin, nor by those made to the eyes. However, there was a trend for slower responses when the faces lacked corneal reflections and there was also a trend for faster (not slower) responses when the faces had the skin smoothened, both in a subtle and in a substantial manner. With none of these trends in the response time data being significant, we will refrain from discussing these trends or their potential causes.

## General discussion

Previous studies have found that memory for virtual faces is worse than for photographic images of humans (Balas & Pacella, 2015; Crookes et al., 2015). The reasons are, however, poorly understood. Previous work also showed that more broadly there exist processing differences between virtual and real faces (Farid & Bravo, 2012; Vaitonytė et al., 2021) and that these differences can be associated with the appearance of the eyes and skin (Balas & Tonsager, 2014). Computational and experimental work revealed that specific features of the eyes and skin might be responsible for the discrepancies in perception between virtual and real faces, in that faces lacking corneal reflections and having reduced skin contrast, i.e., less detail, are judged less human-like (Vaitonytė et al., 2021). However, it remained unclear what the role of corneal reflections and skin contrast is in the context of a more general cognitive task, such as a memory task. Remembering the face involves a perceptual component (Herweg et al., 2020), i.e., the face first needs to be perceived—the information needs to be extracted from a perceptual stimulus—before it can be encoded into memory.

The current memory experiments employing virtual faces (available from companies developing such faces) as well as real human faces (acquired under standardized conditions and manipulated) demonstrated that increased corneal reflections and skin contrast in a face yielded better memory for that face. Experiment 1 showed that the virtual faces that had a higher number of corneal reflections and a higher skin contrast were more likely to be remembered than the virtual faces that had reductions in these features. Experiment 2 allowed us to systematically look at the effect of each predictor using the eye conditions (corneal reflections present vs. corneal reflections absent) and the skin conditions (skin natural vs. skin smoothened subtly vs. skin smoothened substantially). Results showed that the absence of corneal reflections had a statistically significant and negative effect on memory recognition accuracy; however, this was not the case for a smoother skin, which only showed a trend in the predicted direction. This means that the faces with smoother than normal skin tended to be remembered worse but not significantly so. The findings from both experiments provided convergent evidence that the preservation versus the violation of the two features impacts the ability to remember the face. More broadly, these findings suggest that the appearance of eyes and the appearance of skin are relevant to both subjective perceptual decisions when people evaluate the human-likeness of the face and the retrieval of faces from memory.

The two experiments are complementary because they bring corresponding evidence from different contexts. The virtual faces in Experiment 1 are not manipulated and are available in the industry. The human faces in Experiment 2 are not available in the industry but provide a highly controlled set of images.

Due to the nature of how the virtual faces were collected, we were not able to control for any extraneous factors, e.g., distinctiveness and attractiveness, which may influence face memory (Valentine, 1991). Although it seems unlikely that distinctiveness influenced the results by visual inspection of the virtual faces, we cannot rule out this possibility. We compensated for this caveat in Experiment 2 by having a set of faces that were comparable in perceived distinctiveness, attractiveness, and low-level characteristics (i.e., luminance). Future studies may still want to employ a set of virtual faces (e.g., obtained via 3D scanning) and a set of photographic images matched on identity which would enable both the examination of the effect of corneal reflections and skin contrast on memory and a direct comparison between virtual and real faces regarding the memory recognition accuracy. Another valid future direction, more broadly for psychology research, is understanding how skin contrast may vary depending on the face ethnicity and whether this influences the ability to memorize different faces.

In general, examining the role of skin surface information and the eyes in face processing is not new. Especially, skin surface cues have received attention from researchers. Various pieces of evidence show that skin texture is important for face representations of age (Lai et al., 2013), sex (Bruce et al., 1993), race (Bülthoff et al., 2021), discriminating between familiar and unfamiliar faces (Rogers et al., 2022), as well as in making social evaluations, e.g., attractiveness (Jaeger et al., 2018). For instance, recently Bülthoff et al. (2021) demonstrated that it is possible to change the perception of race having exchanged the skin surface details, as well as exchanging the eyes between the Asian and Caucasian faces. Despite this, until recently there has been only scant research on how the skin and the eyes might be implicated in the perception of *human-likeness* (Cheetham et al., 2013; MacDorman et al., 2009), and which specific aspects of the facial appearance are relevant when judging the face as human-like or not (Vaitonytė et al., 2021).

Faces with smooth skin, either obtained via FaceGen or image editing (as was done in this study), show less individuating information (Crookes et al., 2015), making it difficult for them to be memorized. This idea can be illustrated with research that used facial averaging, a technique in which multiple facial images are superimposed onto each other to produce an average image (Sutherland et al., 2017). The faces constructed using this technique come across as attractive and even create the illusion of being familiar, yet they are difficult to remember and distinguish from each other due to a lack of distinctive features (Unnikrishnan, 2009). Of note is, however, that facial averaging reduces distinctiveness both at the level of face structure and skin surface texture.

Corneal reflections are relatively unexplored when it comes to face processing and thus the explanation why the absence of corneal reflections disrupts face memory is at first unintuitive. While there is literature showing that the eyes are the feature that receives a disproportionately large amount of visual attention within the face (Rogers et al., 2018), and provide a wealth of information for social coordination in communicative settings (Hessels, 2020; Ho et al., 2015), such cognitive/motivational explanation as to why people look at the eyes is likely incomplete. As shown by research using eye tracking, people show most interest in visual stimuli that present both social information and visually salient information (Rubo & Gamer, 2018). Regarding corneal reflections, a plausible explanation with respect to their importance might be related to the way the eyes look physically. There is evidence that the eyes are visually cueing attention, even when they are located not on the head but elsewhere in the body (Levy et al., 2013). Moreover, the characteristic appearance of the eyes, i.e., the contrast that is created between the sclera and the darker-colored iris and pupil (Kobayashi & Kohshima, 1997) may reflect a broader sensitivity of the human visual system to contrast polarity relationships in the face (Gilad et al., 2009). Contrast is also a feature that is used in face recognition systems (Viola & Jones, 2001). In humans, the sensitivity to contrast polarity emerges very early on in development since 5- to 6-month-old infants are already sensitive to contrast reversal in the eyes (Ichikawa et al., 2013).

Prior studies found that contrast is crucial for typical face processing. However, to our knowledge no previous work has linked corneal reflections to the susceptibility of humans to contrast polarity despite that the mechanism at play for both might be similar. While it is unclear to what extent corneal reflections appear robustly in facial images, it can be suggested following recent work that examined the faces generated using Generative Adversarial Networks (GANs) (Hu et al., 2021) that corneal reflections represent a fairly stable feature. It was shown that the similarity of corneal reflections across both eyes was diagnostic when automatically discriminating between real facial images and those created using GANs (Hu et al., 2021). This result permits speculating that corneal reflections occur reliably, at least in the faces depicted in frontal view, otherwise corneal reflections would not be incorporated into the faces produced using GANs, albeit in their imperfect form. Taken together, the eyes as a feature, and within the eyes, corneal reflections represent the cue that is exploited by the visual system for face processing and subsequently memory.

From the practical side, identifying important facial features for typical face processing has implications for real-world applications, such as the development of Intelligent Virtual Agents, and more generally, virtual renditions of real people. Our research identifies facial features that need to be rendered in a detailed manner to create naturally looking artificial faces. Given the role of accumulated experience on face representations (Caharel et al., 2002), it is natural human faces that show typical face processing, whereas the faces that come across as unnatural exhibit deficient processing and are remembered poorly. Such faces are also not likely to evoke animacy perception (Wheatley et al., 2011). Perceiving an entity as an animate is important for sustaining in-depth processing and engagement. For example, the faces of dolls do not sustain continued perceptual processing as opposed to natural human faces as revealed using electroencephalography (Wheatley et al., 2011). Other research that employed artificial agent stimuli, including virtual agent faces, and functional magnetic resonance imaging, similarly showed that there exist neural differences between the processing of faces of humans and the faces of artificial agents, with reduced responses for the latter in the face-selective brain region, the fusiform face area (FFA) (Cheetham et al., 2011; Rosenthal-von der Pütten et al., 2019; Wang & Quadflieg, 2015). In other words, the FFA, which is the brain area that is specialized for face processing is less active when the facial stimuli are not full-blown faces.

The real extent of the implications on users of reduced neural activation in response to *almost but not quite real* faces remains unclear. In fact, neuroscientific evidence for the existence of the uncanny valley (Mori, 1970/2012) is slim (Rosenthal-von der Pütten et al., 2019). Despite this, there is robust evidence that humans have high standards for what they consider human-like (Looser & Wheatley, 2010), and that the entities that are higher in perceived human-likeness/realism engender more positive social interactions compared to those that are lower in human-likeness/realism (Yee et al., 2007). Having virtual agents with natural faces may thus benefit future applications in scenarios, such as e-commerce (Etemad-Sajadi, 2016), healthcare (Robinson et al., 2014), and education (Belpaeme et al., 2018). In such settings, face-to-face interactions are desirable and need to be supported by the extraction of social cues from faces. More generally, our results may also be applicable, although this has to be ascertained directly, to immersive virtual reality contexts, especially those that are aimed at simulating agents and their socio-communicative behaviors (Roth et al., 2019).

Regardless of the method that is used to synthesize the face, e.g., FaceGen or 3D scanning, there is still a need to test which specific facial characteristics in stimuli are associated with observers’ perception and memory. In fact, using various methods to synthesize the face and different face stimuli permits a thorough understanding of the cognitive processes involved in face processing. While computational advancements allow for automatic classification of images into real and artificial through, for example, neural networks (Liu et al., 2020), the process behind the classification decision is typically not transparent (Zhang et al., 2021). This warrants an experimental investigation to uncover the stimulus characteristics that drive human responses to faces, the knowledge of which may benefit researchers working at the intersection of virtual agent development and psychology. Our work shows that at least two of these facial parts (i.e., cornea and skin) need to be modeled with high fidelity or else the generated faces risk engendering deficient processing (cf. Egger et al., 2020). More broadly, those who would like to use artificial faces as stimuli in cognitive psychology research should take into account that the quality of the generated face will influence how it is processed.

As any other study, this study also has its limitations. First, we only had a small number of facial images of virtual agents that were collected from the Internet. Collecting the virtual face images meant that we had a heterogenous set of stimuli in terms of the lighting conditions under which the images were produced, the age of the faces, etc. Similarly, the fact that it was a small number of images that needed to be memorized in Experiment 1 might explain an overall good memory accuracy with respect to the virtual faces. In Experiment 2, we, however, had a larger number of facial images and a systematic approach to manipulate corneal reflections and skin smoothness. Secondly, the current study used static facial images, and thus we are not able to directly extrapolate the results to dynamic contexts which are more ecologically valid than a static context. Despite this, the current study has laid the foundation for follow-up experiments with dynamic 3D faces. Dynamics is a fruitful future direction as it would enable us to examine the processing of virtual faces under conditions that approach real-life settings.

## Conclusions

Naturalistic facial appearance of virtual agents, or a lack thereof, affects how users perceive and engage with these agents. The results in this work showed that the retrieval of faces from memory is influenced by the degree of corneal reflections being present in the eyes, and—albeit to a lesser extent—skin contrast, with higher skin contrast (more detailed and thus natural skin appearance) indicative of better face memory. The current findings are useful to cognitive and social psychologists as well as to developers wishing to create artificial faces that are engaging and emulate naturalistic human appearance.

## Data Availability

All data underlying this research are made available on the Open Science Framework and can be accessed by visiting https://osf.io/5zc9b/.
